# Impact of gadolinium-based MRI contrast agent and local anesthetics co-administration on chondrogenic gadolinium uptake and cytotoxicity

**DOI:** 10.1016/j.heliyon.2024.e29719

**Published:** 2024-04-15

**Authors:** Alexander Zimmerer, Frank Schulze, Sebastian Gebhardt, Katrin Huesker, Dirk Stobbe, Daniel Grolimund, Bernhard Hesse, Georgi I. Wassilew, Janosch Schoon

**Affiliations:** a Center for Orthopaedics, Trauma Surgery and Rehabilitation Medicine, University Medicine Greifswald, Germany; b Diakonieklinikum Stuttgart, Department of Orthopaedic and Trauma Surgery, Orthopädische Klinik Paulinenhilfe, Stuttgart, Germany; c Immunology Department, Institute for Medical Diagnostics (IMD), Berlin, Germany; d Swiss Light Source, Paul Scherrer Institute, Villigen, Switzerland; e Xploraytion GmbH, Berlin, Germany; f ESRF-The European Synchrotron, Grenoble, France

## Abstract

The gadolinium-based contrast agent DOTA-Gd is clinically used in combination with local anesthetics for direct magnetic resonance arthrography. It remains unclear whether gadolinium uptake into cartilage is influenced by co-administration of bupivacaine or ropivacaine and whether DOTA-Gd alters their chondrotoxicity. Gadolinium quantification of chondrogenic spheroids revealed enhanced gadolinium uptake after simultaneous exposure to local anesthetics. Analyses of the spatial gadolinium distribution using synchrotron X-ray-fluorescence scanning indicates gadolinium exposed chondrocytes. In vitro exposure to DOTA-Gd does not alter viability and proliferation of human chondrocytes and the chondrotoxic potential of the anesthetics. Reduced viability induced by ropivacaine was found to be reversible, while exposure to bupivacaine leads to irreversible cell death. Our data suggest that ropivacaine is more tolerable than bupivacaine and that DOTA-Gd exposure does not alter the cytotoxicity of both anesthetics. Enhanced gadolinium uptake into cartilage due to co-administration of anesthetics should find attention.

## 1 Introduction

Femoroacetabular impingement syndrome (FAIS) is a motion-dependent clinical disorder associated with a triad of hip pain, signs of restricted motion, and a conflicting contact between the proximal femur and the acetabular rim [1]. The radiographic assessment is one of the keystones of hip assessment in FAIS. An anteroposterior pelvic radiograph and a Dunn 45° view are recommended for the initial radiographic assessment, while magnetic resonance imaging (MRI) is applied to evaluate soft tissue structures [2]. In this context, direct magnetic resonance arthrography (dMRA) has emerged as the gold standard for visualization of intra-articular lesions and is superior to conventional magnetic resonance imaging and indirect MRA in a meta-analysis [3].

In dMRA, anionic Gadolinium (Gd) contrast agents are used for the visualization of abnormalities in the cartilage layer of the hip joint [4]. Cartilage has a high content of water (80 %) that is associated with an extracellular matrix comprised of type II collagen and proteoglycans. In hyaline cartilage, aggrecan is the major component of proteoglycan side chains. Aggrecan itself also contains branched carbohydrate side chains that are comprised of the glycosaminoglycans (GAG) chondroitin sulfate and keratan sulfate [5]. These sulfated side chains are responsible for the overall negative charge of proteoglycans and are involved in the reversible binding of water thereby facilitating the high resistance for compression in cartilage [6]. Healthy cartilage does therefore repel anionic contrast agents, while in compromised cartilage contrast agents will accumulate due to a loss in negatively charged proteoglycan side chains.

In clinical practice, it is common to combine infiltration of the contrast agent with local anesthetics to potentially reduce pain due to increased volume in the hip joint [7,8]. However, it has been shown in recent years that local anesthetics can have a toxic effect on chondrocytes and cartilage [[9], [10], [11]]. First evidence of chondrotoxicity accumulated after the introduction of automated pumps for pain management after arthroplasties of the shoulder. Here, unusually high numbers of cases of postoperative glenohumeral chondrolysis were observed, indicating that repeated doses of anesthetics are followed by pathologic changes in cartilage [[12], [13], [14], [15], [16]].

Subsequent in vitro investigations on human cartilage explants and isolated chondrocytes corroborated the finding that anesthetics used in the local pain management of joints are chondrotoxic [11,[17], [18], [19], [20], [21]]. In this context, it is noteworthy that the exact degree of toxicity depends on the anesthetic used, the dosage, the number of doses and the time of exposure. Yet, it is still not clear if a single intraarticular injection of anesthetics can be regarded as safe or if this would result in detrimental long-term effects on the cartilage [[9], [10], [11]]. The applied dose is most often reported in percentage for the anesthetic solution used [9]. This is, however, not necessarily representative for the amount of anesthetic compound effectively reaching the cells, i.e. the cellular dose. While all of the mentioned studies are vital for confirming and characterizing the chondrotoxic effect of bupivacaine and ropivacaine, it remains unclear if a single injection with clinically relevant doses into the joint capsule is safe or not.

In addition, there is some evidence that contrast agents containing Gd may also have a potential chondrotoxic effect. Gd-based contrast agents are generally thought to be safe in clinical applications and are rapidly cleared from the body by renal excretion [22]. While low-grade exposure may persist for weeks after contrast agent deliverance [23] and some evidence for systemic toxicity has been described in literature, detrimental effects of Gd-based contrast agents have been mostly attributed to compromised renal function that hamper their excretion [24]. In a clinical setting, evidence for chondrotoxicity of Gd has not been reported yet. A small number of studies have investigated Gd-based contrast agents for their potential to induce chondrotoxic effects in vitro [[25], [26], [27]] and in animal experiments [28]. However, hints on chondrotoxicity only appeared when applied in supraphysiological concentrations or for exposure times that exceed those common in clinical practice. These studies did not investigate if toxic effects are associated with changes in Gd uptake, which would be a prerequisite for direct exposure of chondrocytes embedded in the cartilage matrix. Until today, it also remains an open question whether co-exposure to local anesthetics and associated chondrotoxicity leads to altered uptake of Gd into cartilage matrix. Taken together, there is a lack of available knowledge on how cartilage uptake of Gd and potential chondrotoxic effects are affected when contrast agents and local anesthetics are combined.

Therefore, this in vitro study aimed to (1) quantitatively analyze Gd uptake into chondrogenic matrix following exposure to a routinely used Gd-based contrast agent (DOTA-Gd) if co-administered with bupivacaine or ropivacaine and to (2) assess possible cytotoxic effects of bupivacaine or ropivacaine in combination with DOTA-Gd.

It was hypothesized that the Gd uptake is enhanced due to co-administration of local anesthetics since they exhibit detrimental effects on chondrocytes and subsequently damage the cartilage matrix.

## 2 Material and methods

### 2.1 Patient recruitment and sample harvest

Cartilage samples from four female and three male patients with a mean age of 38.0 years (range = 18.4–59.4 years) were harvested from the lateral femoral condyle in the course of knee arthroscopy following traumatic cartilage lesion. Ethics approval (BB 009/21) was obtained from the local independent ethics committee of the University Medicine Greifswald according to the World Medical Association Declaration of Helsinki. All patients provided written informed consent for the collection and analysis of cartilage samples and associated cells, as well as for the publication of anonymized patient data, including age and gender.

### 2.2 Cell isolation, cultivation, and cryopreservation

The cartilage samples were cut into pieces of <1 mm^3^ with a scalpel. After transfer of 25 mg/cm^2^ to 6-well tissue culture plates, the samples were cultured in expansion medium (EM) consisting of low glucose DMEM (PAN Biotech) supplemented with 10 % fetal bovine serum (FBS Superior, Sigma-Aldrich), 100 U/ml penicillin, 100 μg/ml streptomycin (Thermo Fisher) and 2 mM L-alanyl-l-glutamine (GlutaMAX, Thermo Fisher). The first media change was performed at day four (d4) after seeding. Subsequent media changes were performed twice a week. At day 14–16 of primary cell culture, the cells reached 80 % confluence, were detached with trypsin (PAN Biotech), counted using the TC20 automated cell counter (Bio-Rad) and re-seeded at a cell density of 1.000 cells/cm^2^. 1 × 10^6^ cells were cryopreserved at cell culture passage two in 500 μl freezing media consisting of low glucose DMEM supplemented with 12.5 % human serum albumin (Albiomin, Biotest AG) and 10 % Dimethyl sulfoxide (DMSO, Applichem). The cell suspension was stored at −80 °C in CoolCell™ freezing containers (Corning) to ensure a repeatable −1 °C/min cooling rate before storing the cells in liquid nitrogen.

### 2.3 Exposure to DOTA-gadolinium and local anesthetics

In vitro exposure to DOTA-Gd (ARTIREM 0,0025 mmol/ml, Guerbet), ropivacaine (ROPIvacain® 75 mg/10 ml, B. Braun) and bupivacaine (Bupivacain 0,5 %, JENAPHARM) was realized by treating chondrogenic spheroids and chondrocytes with different amounts of the pharmaceutical products which are routinely used at our department in the course of MRI for visualization of intra-articular lesions. The three pharmaceutics contain H_2_O, NaCl and sodium hydroxide/hydrochloric acid for pH adjustment. ARTIREM contains meglumine as an excipient. Medium for in vitro exposure of chondrogenic spheroids was prepared by diluting the respective pharmaceutical product in high glucose (4.5 g/L) DMEM (Sigma-Aldrich) supplemented with 1.25 mg/ml human serum albumin. Medium for in vitro exposure of chondrocyte monolayers (cell viability and proliferation) was prepared by diluting the respective pharmaceutical product in low glucose DMEM supplemented with 10 % FBS, 100 U/ml penicillin, 100 μg/ml streptomycin and 2 mM L-alanyl-l-glutamine. A dilution of 1:5 was chosen to reach a clinically relevant dose (high dose) since this dilution emulates the pharmaceutical product to synovial fluid ratio following intra-articular injection. The 1:5 dilution corresponds to concentrations of 500 μmoL/l DOTA-Gd, 1000 μg/ml bupivacaine and 1500 μg/ml robivacaine. To determine the effective doses, additional 10-fold (medium dose) and 100-fold (low dose) lower concentrations were used. The resulting doses should be considered as doses that may occur early in the course of elimination after injection and might also be representative for exposure in deeper cartilage layers. The respective concentrations, amounts of substance and amounts of substance per cell of each experimental condition are listed in Table 1. NaCl 0.9 % (Fresenius) was used to balance the volume when cells were exposed to either contrast agent or local anesthetic and as drug vehicle to ensure constant concentrations of media supplements across the different groups.

**Table 1:** Implemented dosimetry for in vitro exposure to DOTA-Gd and/or local anesthetics.

assay	cell viability			proliferation		
dose	higha)	mediumb)	lowb)	higha)	mediumb)	lowb)
cells/well [N]	12,000	12,000	12,000	2400	2400	2400
medium/well [μl]	200	200	200	200	200	200
c DOTA-Gd [μM]	500	50	5	500	50	5
n DOTA-Gd [nmol]	100	10	1	100	10	1
n DOTA-Gd/cell [pmol]	8.3	0.83	0.083	41.7	4.17	0.417
c bupi [μg/ml]	1000	100	10	1000	100	10
m bupi [μg]	200	20	2	200	20	2
m bupi/cell [ng]	16.7	1.67	0.167	83.3	8.33	0.833
c ropi [μg/ml]	1500	150	15	1500	150	15
m ropi [μg]	300	30	3	300	30	3
m ropi/cell [ng]	25	2.5	0.25	125	12.5	1.25

Abbreviations: DOTA-Gd, DOTA-gadolinium; bupi, bupivacaine; ropi, ropivacaine.

a) Dose considered most clinically relevant and used for in vitro assays of cell viability and proliferation in cells from six donors.

b) Doses that were additionally used for the assessment of effective doses (Figs. S1 and S2) using cells from one donor (six technical replicates).

### 2.4 Chondrogenic spheroid culture - exposure in 3D

Chondrocytes from cell culture passage two were thawed, cultured until they reached 80 % confluence and detached for subsequent spheroid culture in cell culture passage four. To this end, 3 × 10^5^ cells were centrifuged in conical 15 ml tubes at 400×*g*. Chondrogenic matrix formation was induced by culturing the spheroids in 500 μl high glucose DMEM supplemented with 100 U/ml penicillin, 100 μg/ml streptomycin, 2 mM L-alanyl-l-glutamine, 10 ng/ml recombinant human transforming growth factor beta 1 (Biolegend), 100 nM dexamethasone, 50 μg/ml l-ascorbic acid 2-phosphate sesquimagnesium salt hydrate, 40 μg/ml l-proline (all Sigma-Aldrich), 1 mM sodium pyruvate (PAN-Biotech), 6.25 μg/ml insulin-transferrin-sodium selenite (Sigma-Aldrich) and 1.25 mg/ml human serum albumin and 5.35 mg/ml linoleic acid (Sigma-Aldrich). Medium exchange was performed twice a week. After 21 days of 3D culture, the spheroids were exposed for 24h to 500 μl EM containing the concentration applied in the high dose group (Table 1). Phase contrast microscope images of n = 3 spheroids were taken at a hybrid microscope (Rebel, ECHO) prior to area quantification of the cross sections using ImageJ. Spheroids used for Gd quantification by mass spectrometry were transferred to 1.5 ml tubes and centrifuged for 30s at 400×*g* before residual medium was removed. These spheroids were stored at −80 °C, while the spheroids used for spatially resolved Gd quantification were embedded in optimal cutting temperature (O.C.T.) compound (Tissue-Tek) and subsequently stored at −80 °C. Spheroids used for histological evaluation were fixed for 1h in Phosphate buffered saline (PBS, Bio&Sell GmbH) containing 4 % v/v formaldehyde (Carl Roth). For histology, 5 μm sections were prepared, mounted on glass slides, subsequently washed with PBS, dehydrated by gradient ethanol/xylene (Th. Geyer), embedded in paraffin, dewaxed with xylene and stained with alcian blue/nuclear fast red. In brief, after rehydration, the sections were acidified for 10 min with 3 % acetic acid and stained for 30 min with 1 % alcian blue solution in 3 % acetic acid (Morphisto). After washing with deionized water, the sections were stained for 5 min with 0.1 % nuclear fast red-aluminum sulfate solution (Carl Roth). Following dehydration and mounting, brightfield imaging of the stained sections was performed at the hybrid microscope.

#### 2.4.1 Gadolinium quantification in 3D spheroids

Spheroids were thawed and enzymatically digested at 65 °C under constant agitation at 500 rpm for 48h in 1 ml of 250 mM phosphate buffer containing 25 mM EDTA (Thermo Fisher) and 1.5 U papain from papaya latex (Sigma-Aldrich). Samples were diluted 1:20 in 1 % HNO_3_ (Nitric acid 65 % suprapur, Sigma-Aldrich) and subsequently analyzed in collision/reaction cell mode by inductively coupled plasma mass spectrometry (ICP-MS, ICapQ, Thermo Fisher), using external and internal standard calibration.

#### 2.4.2 Synchrotron X-ray-fluorescence scanning of 3D spheroids

A 10 μm cryosection was placed between two pieces of a 4 μm thin ultralene foil (Spex SamplePrep) and mounted on custom-made sample holders. Micro x-ray-fluorescence (μXRF) cartography was performed at the microXAS beamline of the Swiss Light Source (Paul Scherrer Institute). In brief, monochromatic excitation radiation of 9.8 keV (above Zn K-edge) was selected using a double crystal monochromator (DCM) equipped with Si(111) crystals. The primary beam was focused by two orthogonal reflective mirrors ('KB-geometry') down to a spot size of 1 μm × 2 μm (h x v). XRF spectra were collected using Silicon Drift Diode (SDD) detector systems. Chemical images were recorded in ‘on-the-fly’ mode, typically with a pixel size of 8 μm × 8 μm based on the required large field of view. XRF spectra obtained for each individual pixel were deconvoluted using the software PyMCA [29,30].

### 2.5 Monolayer culture - exposure in 2D

Chondrocytes from cell culture passage two were thawed and cultured in EM to reach 80 % confluence. Following trypsinization, 1.2 × 10^4^ cells were seeded on 48-well tissue culture plates. After obtaining sufficient cell attachment by culturing for 24h in 200 μl EM, the cells were exposed to the different pharmaceutical products according to the dosimetry reported in Table 1 for either 1h or 24h. Subsequent to exposure, cells were washed twice with EM and further cultivated.

#### 2.5.1 Quantification of cell viability in 2D

Determination of cellular metabolic activity as a marker for cell viability via a resazurin-based assay (PrestoBlue, Thermo Fisher) was performed 1h, 4 days (4d) and 8d after exposure according to the assay manual by quantifying the fluorescence intensity (Ex/Em: 560/590 nm) using a multimode microplate reader (m200 pro, Tecan). The medium was exchanged with 200 μl fresh EM following every time point of viability quantification.

#### 2.5.2 Quantification of proliferation capacity in 2D

Chondrocytes from cell culture passage two were thawed and cultured in EM to reach 80 % confluence. Following trypsinization, 2.4 × 10^3^ cells were seeded on 48-well tissue culture plates. After obtaining sufficient cell attachment by culturing for 24h in 200 μl EM, cells were washed with PBS and frozen at −80 °C for later quantification of the baseline DNA content, i.e. for calculation of cell population doublings at later time points. All other culture plates were exposed according to the dosimetry protocol (Table 1) for either 1h or 24h. The individual plates were washed with PBS after cell viability quantification 1h, 4d and 8d after exposure and frozen at −80 °C. The medium of the culture plate seeded for DNA quantification at d8 after exposure was exchanged with 200 μl fresh EM at d4 after exposure. DNA quantification was performed after thawing of all plates using the fluorescence-based CyQuant assay (Thermo Fisher) according to the assay manual using a multimode microplate reader (m200 pro, Tecan). Population doublings were calculated by relating the fluorescence intensities of d4 and d8 to the value of d0 (24h after seeding, prior to exposure) using the following formula: log (value d4 or d8/value d0)/log (2).

### 2.6 Statistical analysis

Descriptive statistics regarding RGB imaging, heatmap imaging and peak spectra of the µXRF data were realized with PyMCA [29]. Data plotting and exploratory data analyses were implemented by GraphPad Prism 8. Cells from one donor were used to generate chondrogenic spheroids and to investigate Gd uptake as a function of exposure to local anesthetics (n = 8 spheroids per group). Cells from n = 6 donors were used to investigate cell viability and proliferation capacity after exposure to DOTA-Gd or local anesthetics. Quadruplicates were employed per donor and condition. Further details on exploratory statistics regarding sample size and statistical tests are reported in figure captions. A p-value <0.05 was considered statistically significant.

## 3 Results

### 3.1 Gadolinium uptake into cartilage matrix exposed to local anesthetics

To analyze if simultaneous exposure to local anesthetics affects Gd uptake, chondrogenic spheroids were generated in vitro and exposed to DOTA-Gd alone or to DOTA-Gd plus local anesthetics. After enzymatic digestion, the Gd content of the individual spheroid lysates was quantified.

**Fig. 1:**
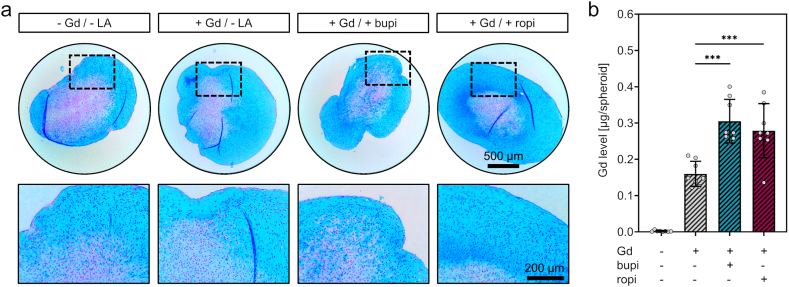
Histological evaluation and quantification of gadolinium levels in chondrogenic spheroids (a) Alcian blue/nuclear fast red stained sections of chondrogenic spheroids indicates successful chondrogenic matrix formation and unaltered chondrogenic matrix after 24h exposure to DOTA-Gd and local anesthetics. (b) Gadolinium level quantification of digested chondrogenic spheroids via inductively coupled plasma mass spectrometry shows enhanced gadolinium levels in spheroids co-exposed to bupivacaine or ropivacaine. [n = 8 spheroids/group generated with cells from one donor; mean ± standard deviation, one-way ANOVA with Bonferroni's post-hoc test; levels of significance: ***p < 0.001]. Abbreviations: LA, local anesthetics; Gd, DOTA-gadolinium; bupi, bupivacaine; ropi, ropivacaine. (For interpretation of the references to colour in this figure legend, the reader is referred to the Web version of this article.)

In order to evaluate successful generation of chondrogenic matrix and to assess the influence of the respective exposure on the integrity of the chondrogenic matrix, one spheroid per group was histologically processed. Histological staining with alcian blue/nuclear fast red revealed no histological differences between groups in terms of abundance of mucins, sulfated GAGs, or of cell density (Fig. 1a). Gd quantification of lysates from n = 8 spheroids per group resulted in the following mean Gd levels [μg/spheroid ± SD]: control, <0.01; DOTA-Gd, 0.16 ± 0.03; DOTA-Gd plus bupivacaine, 0.30 ± 0.06; DOTA-Gd plus ropivacaine, 0.28 ± 0.07 (Fig. 1b). Statistical analysis showed significantly higher Gd uptake into chondrogenic spheroids co-exposed to the local anesthetics, bupivacaine (1.9-fold, p = <0.001) and ropivacaine (1.7-fold, p = <0.001). Each spheroid analyzed was exposed to 500 μl medium with a DOTA-Gd concentration of 500 μmoL/l for 24h. This corresponds to a total dose of 140 μg DOTA-Gd or 39.3 μg Gd per spheroid. The calculation of the proportion of Gd that has been taken up resulted in the following mean percentage uptake [% ± SD]: DOTA-Gd, 0.41 ± 0.09; DOTA-Gd plus bupivacaine, 0.78 ± 0.15; DOTA-Gd plus ropivacaine, 0.71 ± 0.19. These numbers indicate that only a fraction of the available Gd is taken up into cartilage matrix, but this analysis does not allow conclusions regarding Gd concentrations in cartilage matrix compared to the concentration in the administered medium used i.e. regarding Gd accumulation. Thus, the mean spheroid cross section area (4.56 × 10^6^ μm^2^) was calculated by area quantification of cross sections derived from spheroid shapes (Fig. S1). The mean radius of spheroids calculated from the mean spheroid area equates to 1.2 mm and the mean spheroid volume equates to 7.2 μl when approximating spherical shapes. Based on the quantified Gd levels and the approximated spheroid volume, the detected mean Gd concentrations within the DOTA-Gd exposed spheroids were as follows [μg/ml ± SD]: DOTA-Gd, 22.2 ± 4.8; DOTA-Gd plus bupivacaine, 42.4 ± 8.4; DOTA-Gd plus ropivacaine, 38.7 ± 10.4. The concentration of Gd in the administered medium equates 500 μmoL/l (Table 1) or 78.6 μg/ml, respectively. This indicates that the Gd concentrations within all spheroids are lower when compared to Gd concentrations in the surrounding medium and that there is an increased uptake due to co-exposure to local anesthetics but no directional accumulation.

Taken together, these analyses showed that 24h exposure to DOTA-Gd or to DOTA-Gd plus local anesthetics, does not result in histologically detectable differences in the quality of the chondrogenic matrix. Co-exposure to the local anesthetics bupivacaine or ropivacaine results in higher uptake of Gd into chondrogenic matrix.

### 3.2 Spatially resolved detection of gadolinium in cartilage matrix

Quantification of Gd amount in the lysed chondrogenic spheroids clearly showed Gd uptake after exposure to DOTA-Gd. However, based on these analyses, no conclusion can be drawn about the distribution of Gd in the chondrogenic matrix or whether Gd is absorbed only in the edge regions of the chondrogenic spheroid. Therefore, spatially resolved multi-element detection using synchrotron μXRF-cartography of a chondrogenic spheroid exposed to DOTA-Gd and bupivacaine was performed to analyze whether Gd fully integrates into the cartilage matrix and, if so, whether Gd is co-localized with cell and matrix elements.

**Fig. 2:**
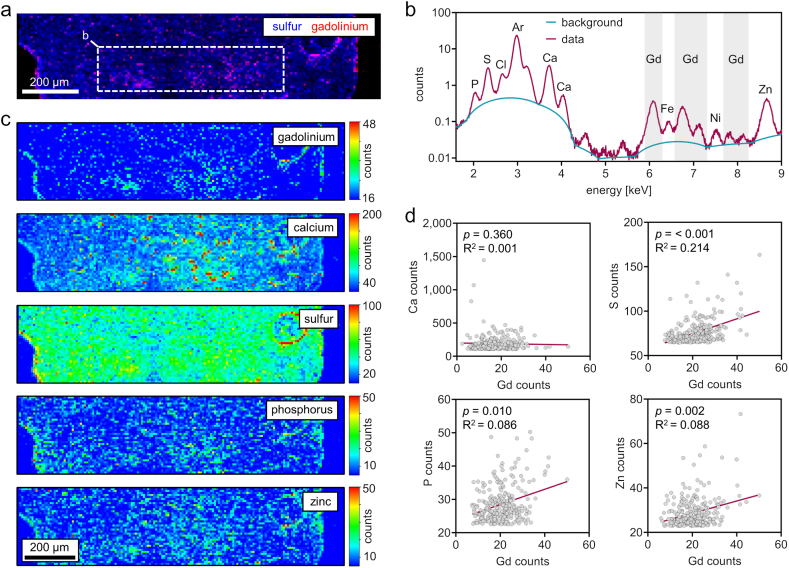
Spatially resolved synchrotron-µXRF-based multi elemental analysis reveals that gadolinium integrates into chondrogenic matrix following 24h exposure to DOTA-GD and bupivacaine (a) Qualitative μXRF map with a pixel size of 8 μm indicates exposure to gadolinium at edge and core regions of a chondrogenic spheroid. (b) Averaged XRF-spectrum of the spheroid inner region clearly indicates the presence of gadolinium (c) Semi-quantitative heat-maps show the elemental composition of the chondrogenic spheroid and indicates sulfur abundance within the chondrogenic matrix and focal calcium, phosphorus and zinc. (d) Linear regression analyses of gadolinium and cell/matrix element counts. Plotted are the counts of all pixels within the upper 95 % quantile of the respective cell/matrix element. Linear regression and correlation analyses of gadolinium counts and element counts shows a correlation of gadolinium counts with the matrix elements sulfur, phosphorus and zinc. [n = 275, two-tailed Spearman correlation].

The spatially resolved analysis clearly showed that Gd is present in all regions of the chondrogenic spheroid section (Fig. 2a). After deconvolution of the XRF spectra of the inner region of the chondrogenic spheroid, the XRF spectrum indicated characteristic X-ray L-lines of Gd at 6.1 keV, 6.7 keV, 7.1 keV, 7.8 keV and 8.1 keV (Fig. 2b). Semi-quantitative heatmaps of the matrix elements sulfur (S) and calcium (Ca) indicated high abundance of these elements in the chondrogenic matrix while S was equally distributed throughout the spheroid and Ca was rather present in hotspots within the core region of the spheroid. The elements phosphorous (P) and zinc (Zn), which are commonly related to cellular structures, were distributed in hotspots throughout the spheroid indicating the presence of cells within the chondrogenic matrix (Fig. 2c). Linear regression analyses were performed to analyze whether the spatial signal intensity of Gd correlates with the signal intensity of matrix or cell elements. The correlation analyses of all pixels with a signal intensity within the upper 95 % quantile of the respective matrix or cell element indicated that Gd signal intensities were significantly correlated with the signal intensities of the matrix-related element S but not with the signal intensities of Ca. In addition, Gd signal intensities were correlated with the signal intensities of the cell-related elements P and Zn (Fig. 2d).

Taken together, the spatially resolved multi-element detection of a chondrogenic spheroid exposed to DOTA-Gd plus bupivacaine indicates that Gd uptake is present in all regions of the chondrogenic spheroid and that cells within the cartilage matrix are exposed to Gd.

### 3.3 Metabolic activity of chondrocytes exposed to DOTA-Gd and/or anesthetics

Human chondrocytes were exposed to clinically relevant concentrations of DOTA-Gd to evaluate whether the observed Gd uptake into cartilage matrix leads to cytotoxicity. Mitochondrial activity as a marker of cell viability was quantified 1h, 4d, and 8d after exposure for either 1h or 24h. Chondrocytes were exposed to bupivacaine, ropivacaine, or to a combination of either anesthetic and DOTA-Gd, to evaluate whether synergistic effects occur in regard to cell viability.

Cells from one donor were exposed to three different doses (Table 1) to assess the effective dose of reduced cellular viability. These analyses revealed that exposure to DOTA-Gd alone at all doses and exposure times does neither lead to a biologically relevant reduction of cell viability nor to additional reduction of cell viability following co-administration of anesthetics. Exposure for 1h to the high dose of bupivacaine did not lead to a reduction of cell viability whereas exposure for 1h to the high dose of ropivacaine led to a significant and reversible reduction of cell viability (Fig. S2a). Following exposure for 24h to the high dose of bupivacaine, a complete loss of cell viability was observed. Exposure for 24h to the medium dose of bupivacaine led to a significant reduction of cell viability 1h after exposure. This effect was reversible as no significant reduction in cell viability was observed at d4 and d8. Exposure for 24h to the high dose of ropivacaine also resulted in significant and reversible reduction of cell viability (Fig. S2b). Phase-contrast microscopy confirmed these results (Fig. S3). Adherent chondrocytes were not observed at d8 after 24h of exposure to high doses of bupivacaine and bupivacaine plus DOTA-Gd, indicating complete loss of viable cells. Nonadherent cells were also observed after exposure to high doses of ropivacaine and ropivacaine plus DOTA-Gd. Yet, in contrast to cells exposed to bupivacaine, an intact confluent cell layer was found.

**Fig. 3:**
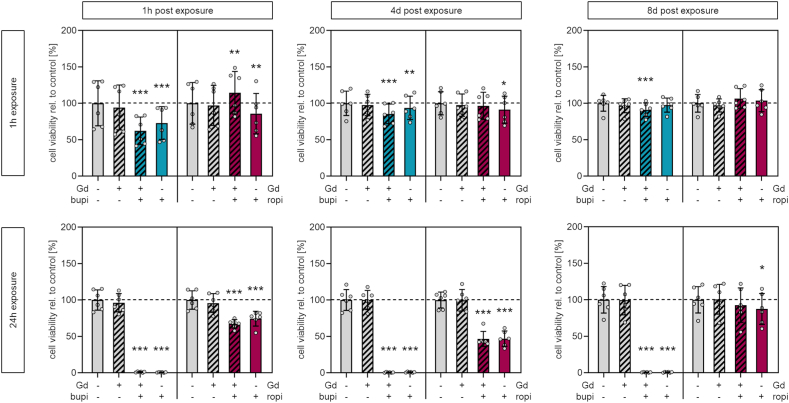
Metabolic activity quantification of chondrocytes exposed to DOTA-Gd and/or locals anesthetics Viability of chondrocytes following 1h exposure (top) and 24h exposure (bottom) to DOTA-Gd, bupivacaine or ropivacaine, or a combination of DOTA-Gd and the respective local anesthetic. Viability was consecutively quantified 1h, 4 days and 8 days after exposure. [n = 6; mean ± standard deviation, Repeated measures one-way ANOVA with Dunnett's post-hoc test; levels of significance: *p < 0.05, **p < 0.01, ***p < 0.001]. Abbreviation: LA, locals anesthetic; Gd, DOTA-gadolinium.

Since significant and biologically relevant reductions in cell viability were only observed after exposure to clinically relevant high doses of local anesthetics and since intra-species variations of these effects and potential donor-individual responses following exposure to local anesthetics and/or DOTA-Gd should be considered, cells from a total of six donors were exposed to high doses. These analyses confirmed that exposure to DOTA-Gd alone does not lead to any significant decrease in cell viability at any time point after exposure for 1h or 24h (Fig. 3). A significant decrease in cell viability was observed following exposure for 1h to bupivacaine plus DOTA-Gd (p = <0.001) and bupivacaine alone (p = <0.001) 1h after exposure [%, mean ± SD]: control, 100.0 ± 30.9; DOTA-Gd plus bupivacaine, 62.3 ± 19.0; bupivacaine, 72.9 ± 22.5. This effect was persistent in both groups (bupivacaine plus DOTA-Gd, p = <0.001; bupivacaine alone, p = 0.007) but less pronounces at d4 after exposure [%, mean ± SD]: control, 100.0 ± 16.8; DOTA-Gd plus bupivacaine, 85.5 ± 13.5; bupivacaine, 93.8 ± 16.1. At d8 after exposure, significantly reduced cell viability was not detectable following exposure to bupivacaine alone but remains significantly reduced (p = <0.001) following exposure to bupivacaine plus DOTA-Gd [%, mean ± SD]: control, 100.0 ± 10.9; DOTA-Gd plus bupivacaine, 91.3 ± 9.3. Exposure to DOTA-Gd plus bupivacaine and bupivacaine alone for 24h resulted in a complete loss of cell viability at all time point. Effects on the viability after exposure to ropivacaine and DOTA-Gd plus ropivacaine were found to be less pronounced. Exposure for 1h to ropivacaine alone resulted in a slight but significant decrease of cell viability 1h after exposure (p = 0.002) and 4d after exposure (p = 0.034) [%, mean ± SD]: control 1h, 100.0 ± 28.6; ropivacaine 1h, 85.8 ± 27.8, control 4d, 100.0 ± 6.5; ropivacaine 4d, 91.3 ± 18.6. Eight days after exposure, reduced cell viability was not observed. Exposure for 1h to DOTA-Gd plus ropivacaine resulted in a slight increase of cell viability, which was found to be significant 1h after exposure (p = 0.001) but not at later time points [%, mean ± SD]: control, 100.0 ± 11.7; DOTA-Gd plus ropivacaine, 114.6 ± 11.9. A significant decrease of cell viability was observed 1h after exposure for 24h to either DOTA-Gd plus ropivacaine (p = <0.001) or ropivacaine alone (p = <0.001) [%, mean ± SD]: control, 100.0 ± 12.7; DOTA-Gd plus ropivacaine, 67.2 ± 5.8; ropivacaine, 74.4 ± 10.1. The reduced cell viability was persistent 4d after exposure [%, mean ± SD]: control, 100.0 ± 11.0; DOTA-Gd plus ropivacaine, 46.6 ± 10.3; ropivacaine, 47.0 ± 10.5. Cell viability of cells exposed to ropivacaine plus DOTA-Gd was not significantly reduced at d8 after exposure, whereas a slight but significant reduction (p = 0.015) in cell viability was still observed after exposure to ropivacaine alone [%, mean ± SD]: control, 100.0 ± 18.1; ropivacaine, 87.5 ± 21.2.

Taken together, bupivacaine is characterized by a higher potential to reduce cellular viability of human chondrocytes if compared to ropivacaine. The distinct reduction of cell viability is not reversible after 24h of bupivacaine exposure, whereas reduced cell viability as a consequence of exposure to ropivacaine is reversible. DOTA-Gd alone has no effect on the cell viability of human chondrocytes and does not alter the chondrotoxic potential of either anesthetic.

### 3.4 Proliferation capacity of chondrocytes exposed to DOTA-Gd and/or anesthetics

DNA quantification of human chondrocytes was performed immediately before and one week after exposure to analyze whether exposure to DOTA-Gd, bupivacaine, ropivacaine, or to a combination of either anesthetic and DOTA-Gd at clinically relevant concentrations leads to a loss of proliferation capacity or cell death. Here, viability was again assessed prior DNA quantification to allow for subsequent normalization of values thus allowing to analyze if reduced viability following exposure to bupivacaine or ropivacaine is a result of reduced cell numbers.

Pre-experimentation using chondrocytes from one donor was performed to identify the effective dose of reduced cell proliferation (Table 1). These analyses revealed that only treatment using the high and clinically most relevant dose led to reduced cell numbers (Fig. S4a) and proliferation rates (Fig. S4b). In brief, exposure to DOTA-Gd alone for either 1h or 24h did not lead to any alteration of cell numbers and proliferation. Exposure to DOTA-Gd plus bupivacaine and bupivacaine alone at the high dose resulted in significantly increased cell numbers and proliferation, whereas exposure for 24 h resulted in cell death indicated by negative proliferation rates. Exposure to high doses DOTA-Gd plus ropivacaine or ropivacaine alone significantly reduced cell numbers and proliferation after either 1h or 24h exposure. To confirm these results and to consider donor-individual responses, cells from six donors were exposed to high doses of local anesthetics and/or DOTA-Gd.

**Fig. 4:**
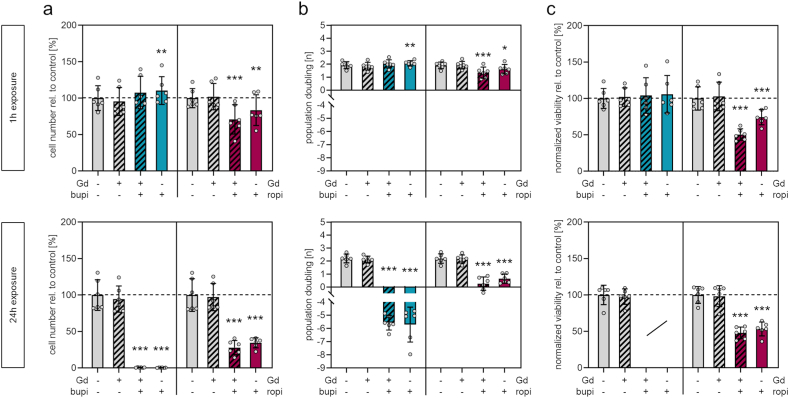
Quantification of chondrocyte numbers, proliferation capacity and cell viability normalized to cell numbers (a) DNA quantification at day 7 after exposure for 1h (top) or 24h (bottom). (b) Calculated cell population doublings at day 7 after exposure for 1h or 24h (c) Cell viability (metabolic activity) at day 7 after exposure for 1h or 24h normalized to cell number (DNA quantification) at day 7 of cells from the respective donor. [n = 6; mean ± standard deviation, Repeated measures one-way ANOVA with Dunnett's post-hoc test; levels of significance: *p < 0.05, **p < 0.01, ***p < 0.001]. Abbreviation: LA, locals anesthetic; Gd, DOTA-gadolinium.

These analyses confirmed that DOTA-Gd alone does not lead to any biologically relevant decrease in cell numbers (Fig. 4a), cell proliferation (Fig. 4b) or viability normalized to cell numbers (Fig. 4c). Exposure to bupivacaine alone for 1h led to a slight and significant (p = 0.009) increase of cell numbers [%, mean ± SD]: control, 100.0 ± 17.0; bupivavaine, 110.4 ± 19.1. No cells were detected after exposure for 24h to DOTA-Gd plus bupivacaine and to bupivacaine alone. In contrast, exposure to ropivacaine for 1h and 24h did not lead to a complete cell loss. Exposure to DOTA-Gd plus ropivacaine and ropivacaine alone for 1h led to significant (p = <0.001 and p = 0.003, respectively) reductions of cell numbers [%, mean ± SD]: control, 100.0 ± 13.2; DOTA-Gd plus bupivacaine, 70.9 ± 20.1; bupivacaine, 83.5 ± 21.1. This effect was found to be more pronounced after 24h of exposure (p = <0.001, both groups) [%, mean ± SD]: control, 100.0 ± 22.4; DOTA-Gd plus bupivacaine, 27.9 ± 9.7; bupivacaine, 34.5 ± 6.9. Calculation of population doublings revealed negative proliferation rates following exposure to DOTA-Gd plus bupivacaine and bupivacaine alone, confirming cell death. Proliferation rates were significantly reduced following exposure for 1h to DOTA-Gd plus ropivacaine (p = <0.001) and ropivacaine alone (p = 0.020) [n, mean ± SD]: control, 1.91 ± 0.26; DOTA-Gd plus ropivacaine, 1.35 ± 0.43; ropivacaine, 1.62 ± 0.36. Exposure for 24h led to a more pronounced reduction of proliferation (p = <0.001, both groups) [n, mean ± SD]: control, 2.19 ± 0.36; DOTA-Gd plus ropivacaine, 0.26 ± 0.51; ropivacaine, 0.65 ± 0.36. Normalization of the cell metabolic activity to cell numbers revealed that the relative cell viability is not reduced following exposure for 1h to DOTA-Gd plus bupivacaine and bupivacaine alone. After exposure for 24h to DOTA-Gd plus bupivacaine and bupivacaine alone, normalization was not possible (no living cells). The relative cell viability after exposure for 1h to DOTA-Gd plus ropivacaine and ropivacaine alone (p = <0.001, both groups) was found to be significantly reduced [%, mean ± SD]: control, 100.0 ± 16.0; DOTA-Gd plus ropivacaine, 50.4 ± 7.9; ropivacaine, 74.3 ± 10.4. Exposure for 24h also led to reduced relative cell viability (p = <0.001, both groups) [%, mean ± SD]: control, 100.0 ± 11.5; DOTA-Gd plus ropivacaine, 47.9 ± 8.1; ropivacaine, 53.3 ± 9.5.

Taken together, exposure to DOTA-Gd alone does not influence human chondrocytes regarding cell number, cell proliferation or cell viability normalized to cell number.

Both anesthetics significantly reduce cell numbers and cell proliferation. Following exposure for 1h, this effect was only detected for ropivacaine whereas a complete loss of cells was detected following 24h of exposure to bupivacaine indicating a higher cytotoxic potential compared to ropivacaine. Calculation of the cell viability relative to the detected cell numbers revealed that exposure to ropivacaine not only leads to reduced proliferation but also to a reduction cell viability of human chondrocytes.

## 4 Discussion

The paramount aim of this study was to analyze if Gd uptake into cartilage is influenced by co-administration of bupivacaine and ropivacaine and if a Gd-based contrast agent alters the chondrotoxicity of the two local anesthetics. We found that Gd uptake is enhanced by co-administration of local anesthetics and that the contrast agent DOTA-Gd did not exhibit any signs of chondrotoxicity alone and did not enhance the toxicity of either bupivacaine or ropivacaine. As reported in literature before, both anesthetics influenced viability of chondrocytes in a dose and exposure time dependent manner [11,20].

The highest dose of anesthetics used in our setup is meant to reflect the exposure that might be encountered by chondrocytes following intra-articular injection into the hip joint using 0.5 % bupivacaine and 0.75 % ropivacaine. The two lower doses thus reflect diminished exposure that might occur in deeper layers of the cartilage layer. The treatment with anesthetics for 1h reflects a clinically relevant exposure time in the joint capsule based on the respective pharmacokinetics of bupivacaine [11,31,32]. Since ropivacaine shares similarities in structure to bupivacaine and has a shorter systemic half-life, it can be assumed that the retention in the joint capsule is comparable if not even shorter [33]. The 24h exposure time is however less representative for the clinical situation after a single injection but might be compared to a continuous exposure due to repeated administration. Therefore, 24h incubation time is rather meant to reflect an experimental setup designed to elicit a clear cellular response that is of relevance for comparing the degree of toxicity for the respective anesthetics and to gain information about underlying mechanisms.

Direct comparison of our data with published literature is hampered by differences in dosages and administration regimes (see also Supplementary Table 1). In this regard, only one study reported all information necessary to calculate a cellular dose [11]. In addition, the anesthetics and their respective dilutions were supplied in 0.9 % sodium chloride only and are not mixed with cell culture media or else prior their application on chondrocytes in published studies [11,[19], [20], [21],^34^]. This is, however, a strong approximation of the injection of local anesthetics into the joint capsule, since the administered solution will mix with synovial fluid, thereby changing the overall concentration. In addition, withdrawing the cell culture media for replacement with sodium chloride solution might introduce effects on the cells on its own. In the present study, we aimed to keep the cells under culture media as a substitute for synovial fluid and to mimic the dilution effect in the experimental setup.

Taking these considerations into account, direct quantitative comparison of our data in regard to cellular dose at 1h exposure time is only possible with the study of Breu et al. while it needs to be kept in mind that the anesthetics were supplied in sodium chloride in the respective work [11]. In the present study, cell viability and proliferation were tested in the concentration ranges of 0.167–83.3 ng/cell for bupivacaine and 0.25–125 ng/cell for ropivacaine. Breu et al. tested in similar concentration ranges with 3.1–50 ng/cell for bupivacaine and 3.1–75 ng/cell for ropivacaine. In their study, the authors also included the anesthetic mepivacaine and finally concluded that ropivacaine was the comparably least toxic compound for the concentration range tested.

In the present study, after a 24h incubation period the effects on viability were reversible for high doses of ropivacaine, while the incubation with bupivacaine at its highest concentration lead to an irreversible loss of cells. In addition, prolongation to 24h of exposure did not severely enhance the effect of ropivacaine on viability and proliferation. In summary, both anesthetics exhibited signs of chondrotoxicity while prolonged incubation with bupivacaine leads to a more toxic response than ropivacaine which is in line with the observation that ropivacaine is less toxic as reported not only by Breu et al. for similar concentration ranges but also by other studies [11,17,20,25].

The comparably lower toxicity of ropivacaine is attributed to the chirality of the compounds with ropivacaine being a pure s-enantiomer, while bupivacaine is a racemate [[35], [36], [37]]. Yet, the exact mechanism of chondrotoxicity of both compounds is still a matter of debate [38]. In general, these local anesthetics block sodium channels, thereby inhibiting the propagation of electric potentials in nerve cells, yet it is assumed that they interfere with all excitable biological membranes within organisms [33]. In myocytes, bupivacaine caused a concentration-dependent mitochondrial depolarization and it was therefore hypothesized that the inhibition of oxidative phosphorylation is the key mediator of bupivacaine toxicity [39]. The assay employed for the quantification of cell viability in the present study measures the activity of oxidoreductases and therefore mitochondrial activity [40]. Indeed, our data shows that the loss of chondrocytes, as demonstrated by a comparably low content of DNA and a negative population doubling, after 24h exposure with the highest dose of bupivacaine is accompanied by a total loss of viability without any recovery. For ropivacaine, proliferation was reduced but not inhibited and effects on the cells’ viability were ameliorated over time, indicating reversibility of effects on mitochondrial activity for this anesthetic. Our data is thus in line with others that attributed the chondrotoxicity of local anesthetics to mitochondrial dysfunction [19,39].

The increase in Gd uptake within the chondrogenic spheroids as a result of co-administration of anesthetics was an unexpected finding, since anionic contrast agents such as DOTA-Gd should not penetrate intact cartilage layers. The increase in Gd uptake observed in this study might be mediated by alterations in the overall charge of either the contrast agent or the cartilage matrix in the spheroid, due to the presence of the cationic anesthetics and other components of their administered formulation such as pH buffers [41]. It would also be conceivable that a direct negative effect of the two anesthetics on cartilage matrix components leads to a higher degree of Gd uptake. This would substantiate the negative effects on proliferation and viability after 24h incubation time observed for both anesthetics tested in our work. It was demonstrated that the amount of GAGs is reduced when the local anesthetic lidocaine is applied, while bupivacaine and ropivacaine seem to have no direct effect on the cartilage matrix [17]. However, incubation times in that study were shorter, with 6h for bupivacaine and 12h for ropivacaine compared to the 24h incubation period applied on the cartilage spheroids in the present work. In addition to the uptake of Gd in the cartilage matrix, a cell-mediated Gd uptake is conceivable. It is likely that metabolically active cells are less prone for Gd uptake than those cells whose membrane integrity is influenced by cytotoxic effects. In the present study, we did not investigate cartilage spheroids for their proteoglycan content in a quantitative matter or cellular Gd uptake in response to cytotoxicity following exposure to local anesthetics. In light of the data presented, further investigations on Gd uptake as a function of altered cartilage matrix and/or cytotoxicity are needed to explain the increase in Gd uptake.

The spatially resolved synchrotron μXRF analysis hints on co-localization of Gd with sulfur groups of the extracellular matrix but also with cells, confirming direct exposure of chondrocytes. This is especially interesting since our data regarding the Gd uptake hints on a twofold increase when anesthetics were co-administered. Albeit this leads to a higher cellular dose of Gd, no synergistic effects on chondrocyte viability or proliferation were detected in our experiments. In this context, it should be noted that a 24h exposure to DOTA-Gd represents an artificial setting, since clearance of contrast agent from the joint capsule is expected to last no longer than 2h as data from animal studies indicates [28]. The chosen in vitro model closely aligns with the clinical scenario regarding intra-articular exposure to Gd-based contrast agents and local anesthetics in the context of dMRA. Nevertheless, the chosen in vitro model holds limitations since the exposed cartilage matrix was generated in vitro. We demonstrated that Gd integrates into the in vitro generated cartilage matrix and co-localizes with chondrocytes. However, these analyses do not provide information about the concentrations to which chondrocytes within cartilage lacunae are exposed. Thus, in the 2D monolayer model, the chosen exposure level might be higher than in the cartilage matrix following intraarticular exposure. However, the selected DOTA-Gd concentrations did not induce direct cytotoxic effects or an influence on the cytotoxicity of local anesthetics. It is unlikely that the increase in Gd uptake due to co-administered anesthetics does increase overall toxicity, yet it remains to be investigated if this interferes with clinical practice regarding MRI-based visualization of articular cartilage.

## 5 Conclusion

We found that ropivacaine is more tolerable than bupivacaine when considering cartilage health in the joint after single injection due to the reversibility of effects on viability. While giving direct advice on dosing ropivacaine remains difficult. It can be concluded from our data that a single injection with the 0.75 % formulation of this local anesthetic in a 1:5 ratio should lead to tolerable concentration levels. We did not find evidence for a synergistic effect on chondrotoxicity when the contrast agent DOTA-Gd was co-administered. We did however observe an increase of Gd uptake into the cartilage matrix when anesthetics were present. This finding needs further investigation since elevated DOTA-Gd levels might influence radiographic results in dMRA leading to false diagnostics. In the next step, the uptake of Gd after anesthetics co-administration and the depth of Gd integration should be analyzed using ex vivo cartilage samples to conclusively assess the potential impact of local anesthetics on MRI diagnostics.

## Funding

This project was partially funded by the AGA-Society for Arthroscopy and Joint-Surgery (10.13039/100011338AZ), the research networks molecular medicine and community medicine and the Clinician Scientist Program at University Medicine Greifswald (SG). We acknowledge support for the Article Processing Charge from the 10.13039/501100001659DFG (German Research Foundation, 491454319) and the Open Access Publication Fund of the University of Greifswald.

## Ethics declarations

This study was reviewed and approved by the independent ethics committee of the University Medicine Greifswald, with the approval number: BB 009/21. All patients provided informed consent to participate in the study.

## Informed consent statement

Informed written consent was obtained from all donors involved in this study.

## Data availability statement

Data associated with this study has not been deposited into a publicly available repository. Data will be made available by the corresponding author upon reasonable request.

## CRediT authorship contribution statement

**Alexander Zimmerer:** Writing – review & editing, Writing – original draft, Funding acquisition, Formal analysis, Conceptualization. **Frank Schulze:** Writing – review & editing, Writing – original draft, Visualization, Formal analysis. **Sebastian Gebhardt:** Writing – review & editing, Funding acquisition. **Katrin Huesker:** Writing – review & editing, Methodology, Investigation. **Dirk Stobbe:** Writing – review & editing, Methodology, Investigation. **Daniel Grolimund:** Writing – review & editing, Software, Methodology, Investigation, Data curation. **Bernhard Hesse:** Software, Methodology, Formal analysis, Data curation, Writing – review & editing. **Georgi I. Wassilew:** Resources, Supervision, Writing – review & editing. **Janosch Schoon:** Writing – review & editing, Writing – original draft, Visualization, Supervision, Project administration, Methodology, Conceptualization, Data curation, Formal analysis, Investigation.

## Declaration of competing interest

The authors declare that they have no known competing financial interests or personal relationships that could have appeared to influence the work reported in this paper.
