# Supplementary material for: Impact of gadolinium-based MRI contrast agent and local anesthetics co-administration on chondrogenic gadolinium uptake and cytotoxicity
Source: Heliyon. 2024 Apr 15;10(8):e29719. doi: 10.1016/j.heliyon.2024.e29719 (PMC11053198; doi:10.1016/j.heliyon.2024.e29719)
Supplement: Multimedia component 1 [file mmc1.docx]

**Supplementary Figure 1**


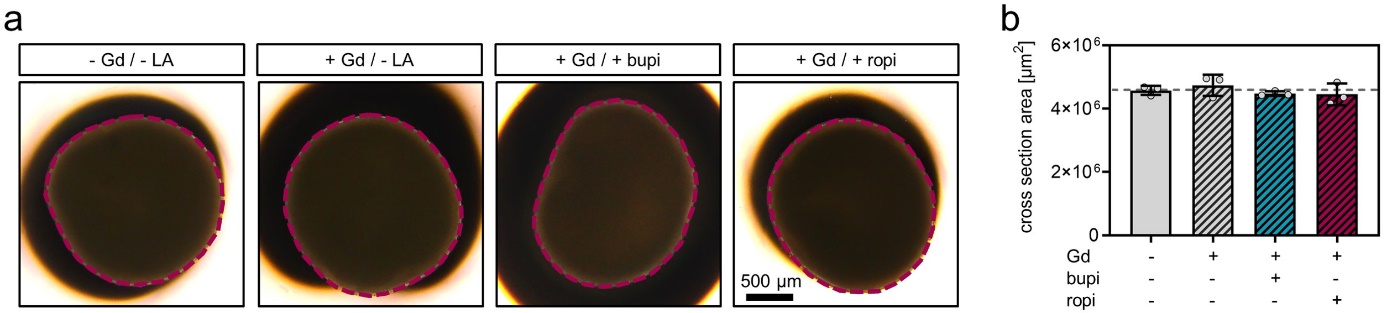


**Fig. S1** Spheroid size is not influenced by exposure to DOTA-Gd or local anesthetics. (a) Representative phase contrast images of chondrogenic spheroids after 21 days of chondrogenic stimulus and subsequent exposure for 24h. The pink lines indicate the shapes of the respective spheroids. (b) Area quantification of cross sections derived from shape indications shows no group differences of spherods’ size [n=3]. Dashed line indicates the mean cross section area of all pallets analyzed (4.56 x 10^6^ µm^2^). Abbreviations: LA, local anesthetics; Gd, DOTA-gadolinium; bupi, bupivacaine; ropi, ropivacaine.

**Supplementary Figure 2**


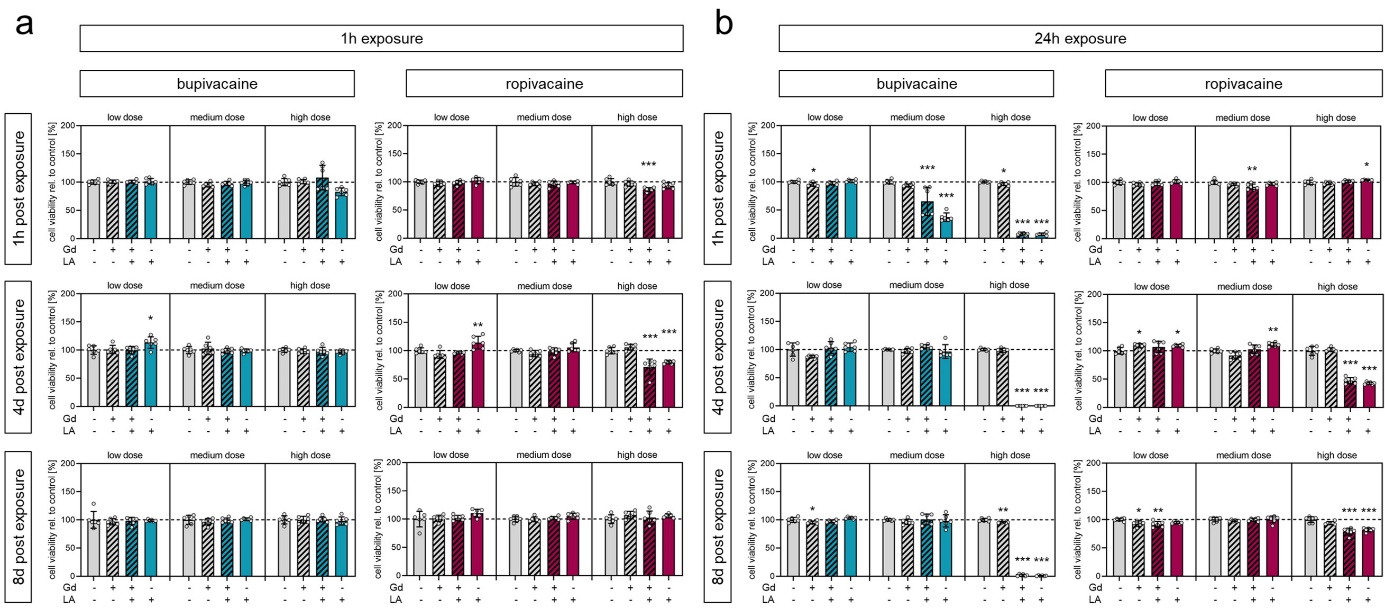


**Fig. S2** Metabolic activity quantification and phase contrast microscopy of chondrocytes exposed to DOTA-GD and/or locals anesthetics. Viability of chondrocytes following 1h exposure (a) and 24h exposure (b) to DOTA-GD, bupivacaine or ropivacaine, or a combination of DOTA-GD and the respective local anesthetic. Viability was consecutively quantified 1h, 4 days and 8 days after exposure and compared to the viability of non-exposed cells. [n=6 technical replicates; mean ± standard deviation, one-way ANOVA with Dunnetts’s post-hoc test (compared to non-exposed cells); levels of significance: *p<0.05, **p<0.01, ***p<0.001]. Abbreviation: LA, locals anesthetic; Gd, DOTA-gadolinium.


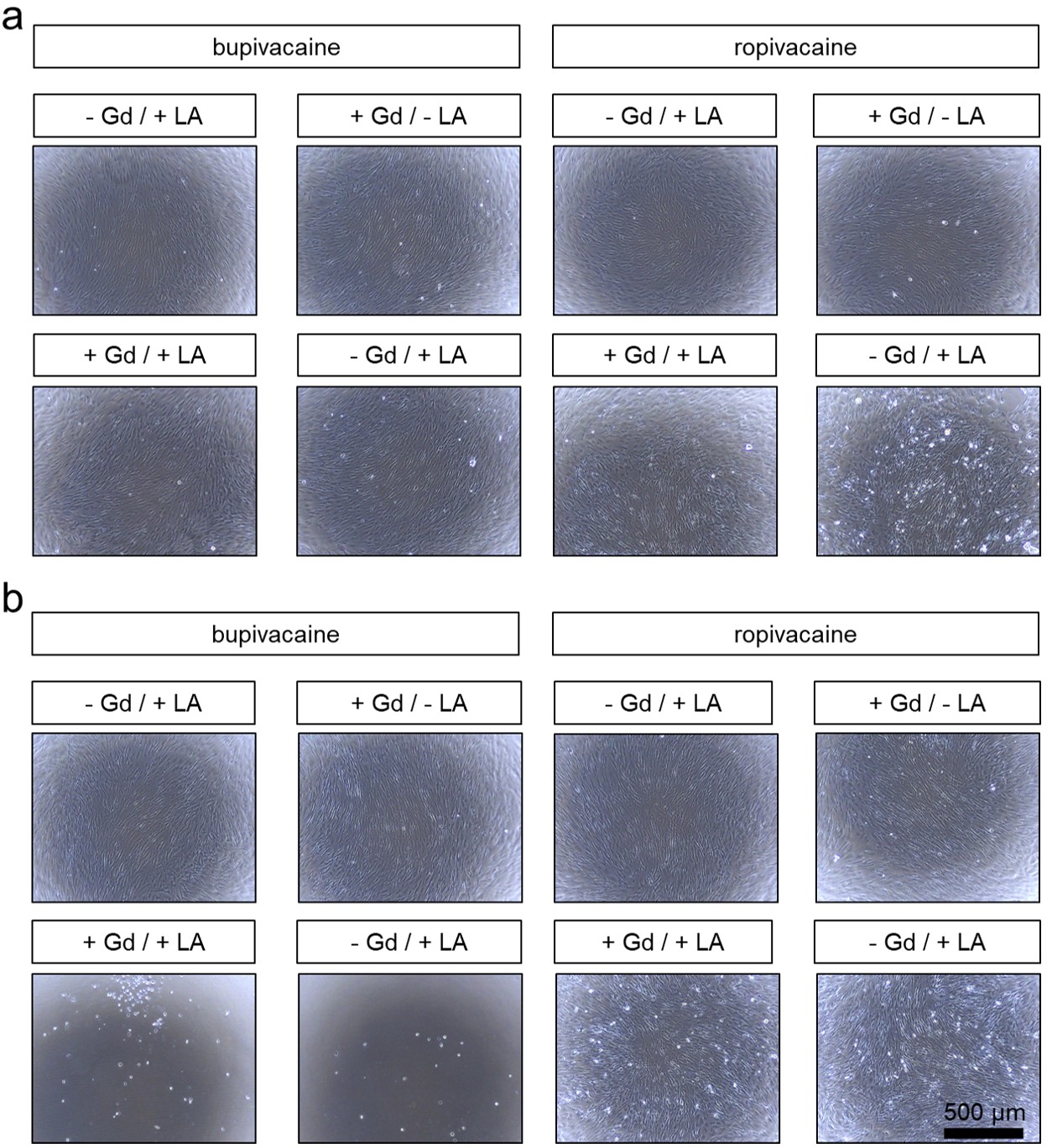


**Fig. S3** Phase contrast microscopy eight days after exposure (high dose) indicates abundance of dead cells following exposure to ropivacaine for 1h (a) and for 24h (b). No viable cells are visible following bupivacaine exposure for 24h with and without co-exposure to DOTA-GD. Abbreviation: LA, locals anesthetic; Gd, DOTA-gadolinium.

**Supplementary Figure 4**


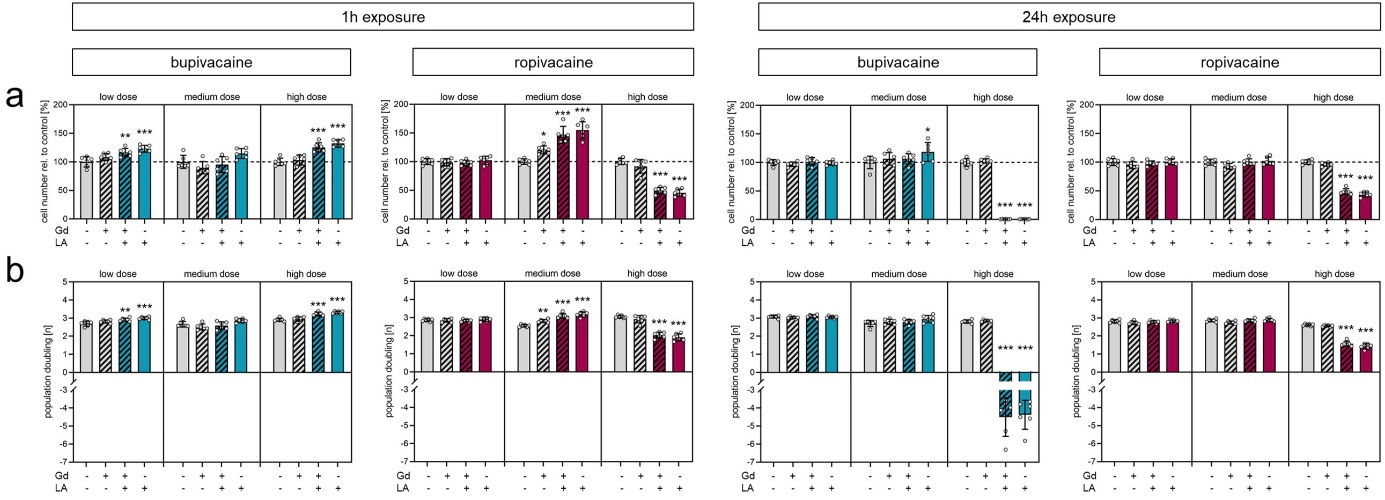


**Fig. S4** Quantification of chondrocyte proliferation reveals enhanced proliferation after short-term exposure to DOTA-GD and/or locals anesthetics and reduced proliferation after 24h exposure to local anesthetics. (a) DNA quantification at day 7 after exposure for 1h or 24h. (b) Calculation of cell population doublings at day 7 after exposure for 1h or 24h reveals negative values following 24h exposure at clinically relevant bupivacaine concentrations indicating cell death. [n=6; mean ± standard deviation, one-way ANOVA with Tukeys’s post-hoc test; levels of significance: *p<0.05, **p<0.01, ***p<0.001]. Abbreviation: LA, locals anesthetic; Gd, DOTA-gadolinium.

**Supplementary Table 1**

**Table S1** Concentrations and doses used for chondrotoxicity testing of bupivacaine and ropivacaine in various in vitro studies.

| **Author** | **Zimmerer et al.** | **Zimmerer et al.** | **Dragoo et al.** | **Dragoo et al.** | **Grishko et al.** | **Piper et al.** | **Breu et al.** | **Chu et al.** | **Syed et al.** |
| --- | --- | --- | --- | --- | --- | --- | --- | --- | --- |
| **year** | 2024 | 2024 | 2012^17^ | 2008^18^ | 2010^19^ | 2008^20^ | 2013^11^ | 2008^33^ | 2011^21^ |
| **exposure time [h]** | 1; 24 | 1; 24 | 6 | 24; 48; 72 | 1 | 0.5 | 1 | 0.25; 0.5; 1 | 0.25 |
| **number of cells [n]** | 12,000 | 2,400 | 500,000 | n.a. | n.a. | 1,000 | 100,000 | n.a. | 1,000 |
| **medium volume [ml]** | 0.2 | 0.2 | n.a. | n.a. | 10 | 0.2 | 1 | 1 | 0.2 |
| **bupivacaine** |  |  |  |  |  |  |  |  |  |
| **c [µg/ml]** | 10; 100; 1,000 | 10; 100; 1,000 | 2,500 | 2,500 | 2,500; 5,000 | 5,000 | 312.5; 625; 1,250; 2,500; 5,000 | 1,250; 2,500; 5,000 | 2,500 |
| **c [ng/cell]** | 0.167; 1.67; 16.7 | 0.833; 8.33; 83.3 | 5 | n.d. | n.d. | 1,000 | 3.13; 6.25; 12.5; 25; 50 | n.d. | 500 |
| **total dose [µg]** | 2; 20; 200 | 2; 20; 200 | n.d. | n.d. | 25,000; 50,000 | 1,000 | 312.5; 625; 1,250; 2,500; 5,000 | 1,250; 2,500; 5,000 | 500 |
| **administration** | mixed with cell culture media | mixed with cell culture media | permanent flow | permanent flow | mixed with saline solution | mixed with saline solution | direct exposure | mixed with saline solution | direct exposure |
| **ropivacaine** |  |  |  |  |  |  |  |  |  |
| **c [µg/ml]** | 15; 150; 1,500 | 15; 150; 1,500 | 5,000 | n.e. | 2,000; 5,000 | 5,000 | 312.5; 625; 1,250; 2,500; 5,000; 7,500 | n.e. | n.e. |
| **c [ng/cell]** | 0.25; 2.5; 25 | 1.25; 12.5; 125 | 10 | n.e. | n.d. | 1,000 | 3.13; 6.25; 12.5; 25; 50; 75 | n.e. | n.e. |
| **total dose [µg]** | 3; 30; 300 | 3; 30; 300 | n.d. | n.e. | 20,000; 50,000 | 1,000 | 312.5; 625; 1,250; 2,500; 5,000; 7,500 | n.e. | n.e. |
| **administration** | mixed with cell culture media | mixed with cell culture media | permanent flow | n.e. | mixed with saline solution | mixed with saline solution | direct exposure | n.e. | n.e. |

Abbreviations: n.a., not announced; n.d., not definable; n.e., not examined
